# Measurement of fecal T3 metabolite levels in sheep: Analytical and biological validation of the method

**DOI:** 10.3389/fvets.2022.1011651

**Published:** 2022-11-25

**Authors:** Valeria Pasciu, Francesca Daniela Sotgiu, Maria Nieddu, Cristian Porcu, Fiammetta Berlinguer

**Affiliations:** ^1^Department of Veterinary Medicine, University of Sassari, Sassari, Italy; ^2^Department of Chemistry and Pharmacy, University of Sassari, Sassari, Italy

**Keywords:** fecal T3 metabolite, ELISA, sheep, reproductive status, lamb, sex

## Abstract

**Introduction:**

Biological sample collection from wild and farms animals is often associated with difficulties related to the handling and restraint procedures, and most of the time it could induce stress, altering the welfare and physiological homeostasis. The analysis of fecal T3 metabolites (FTMs) allows to test samples collected in a non-invasive manner, providing several information about the animal's physiological conditions and the effects related to environmental and nutritional variations. This procedure has found wide application in wild species, but less in domestic ones.

**Methods:**

The aim of this work was to validate the use of an immuno-enzymatic competitive ELISA kit, designed for T3 quantification in human blood serum samples, for the assessment of FTMs in the sheep. For the analytical validation, precision, recovery and parallelism were evaluated; for biological validation the variations of FTMs in relation to age, sex and the physiological status of the animal were determined.

**Results:**

After a verification of the precision (RSD % < 15%), mean recovery (75%) and parallelism (CV% < 10%), the kit was used to measure FTMs in cyclic, pregnant, and early lactating ewes as well as in rams and ewe lambs. The results showed that FTMs concentrations in pregnant ewes were significantly lower (*p* < 0.05) than in cyclic and early lactation ones. Furthermore, there were no significant differences in FTMs levels between ewes and rams, while in lambs FTMs levels were higher than in adults (*p* < 0.001).

**Conclusion:**

In conclusion the present study demonstrates that FTMs can be reliably and accurately determined in sheep feces, using an ELISA kit formulated for human serum T3 assay. The application of this method in the livestock sector could allow to improve our knowledge about the response of animals to different physiological and environmental conditions, and thus assess their welfare.

## Introduction

Thyroid hormones (THs) are known to regulate metabolism, blood pressure, body temperature, and energy expenditure (1–4). As such, they can be considered as indicators of animal metabolic and nutritional status (5) by regulating and stimulating proteins, fat, carbohydrate, and vitamin metabolism (3, 6).

THs are released by the thyroid gland under the regulation of the hypothalamus-pituitary-thyroid gland axis (7). Thyrotropin-releasing hormone (TRH), produced and released by the hypothalamus, stimulate the anterior pituitary gland to secrete thyroid-stimulating hormone (TSH, or thyrotropin) into the bloodstream. In the thyroid follicles gland, TSH stimulates the production of tetraiodothyronine (T4) and triiodothyronine (T3). The major form of THs in the blood is thyroxin (T4), but triiodothyronine (T3) is more biologically active and potent than T4 (8). Most of circulating T3 originates at peripheral levels from T4 conversion, which has a longer blood half-life (9).

The secretion of different quantity of THs is an important adaptive response to changes in physiological status, energy balance and environmental conditions (10). For example, THs concentrations change with age (11, 12), sex (13), and seasons (3, 4), and their secretion can also be affected by other factors such as nutritional condition (11, 14), environmental temperature (15, 16), and reproductive status (12, 17).

THs play a key role in animal's metabolic responses to adapt to changes of external conditions, variations in nutrient requirements and availability, and to homeorhetic changes during different physiological stages. In several ungulates, including sheep (*Ovis* spp.), donkeys (*Equus asinus*) and llamas (*Lama glama*), THs increase in response to cold ambient temperatures (18–20), thus highlighting the critical role of this hormones in the thermoregulation mechanism (2). The study of THs fluctuations is also particularly useful to characterize nutritional stress (3, 21, 22).

Moreover, thyroid activity increases throughout pregnancy (23) and THs act as mediators in this process by directing nutrients and energy to the maternal reproductive tissues and the developing fetus (24). During lactation, adjustments of THs levels are essential to establish the metabolic priority for the lactating mammary gland (25). In human and non-human primates, THs concentrations have indeed been reported to be higher in pregnant and lactating females than in cyclic females (26, 27) and conversely other studies have shown that reduced reproductive success is associated with low THs levels (28).

In livestock, the evaluation of THs fluctuations can help to obtain useful information about animal's response to different physiological and environmental conditions, and hence to assess their welfare.

In both birds and mammals, THs are excreted in the bile (29–31) and their concentrations can thus be determined in feces. In feces THs are found as fecal thyroid metabolites (FTMs), with T3 representing the major metabolite (3). The main advantages of analyzing hormone metabolite levels in the fecal matrix relies to their easy accessibility, to the reduction of the stress related to handling and blood collection procedures, and the variety of hormones that can be measured (3, 21, 32).

This practice, that has found a large use in wildlife species (33–36), still has a minor use in domestic animals.

The validation of the analytical method in a given species is essential to correctly interpret the results found (34, 37). Given these premises, the aim of this work is to validate the use of an immuno-enzymatic competitive ELISA kit, designed for T3 quantification in human blood serum samples, for the assessment of FTMs in the sheep. The same method was recently validated for the measurement of FTMs in European mouflons (*Ovis aries musimon*). This ELISA kit is based on polyclonal antibodies group specific, capable of detecting the predominant fecal metabolites of the parent hormone (35). In this work, we performed both an analytical validation, in terms of parallelism, recovery and precision, and a biological validation, investigating the FTMs changes in relation to age, sex, and the physiological status of the animal.

## Materials and methods

### Materials

Triiodothyronine (T3) standard, ethanol and Phosphate Buffered Saline was obtained from Merck [cod. 642511, 51976, and 806552); T3 ELISA Kit was obtained from Diametra (DiaMetra Srl Management and Coordination: Immunodiagnostic Systems (IDS) Ltd., Boldon, UK, a PerkinElmer Company catalog REF DKO044].

### Animals

The experiment was carried out at Bonassai research station of Agris Sardegna and the Department of Veterinary Medicine at the University of Sassari, located both in north-western Sardinia, Italy. Sarda sheep, used for this study, were selected from the same experimental flock, and they were not used in previous experiments. Animals were fed with concentrate and hay with freely available water and housed in semi-open pens.

Fecal samples were collected from both lambs (40–50 days old; *n* = 10) and adult (3–5 years old) ewes (*n* = 30) and rams (*n* = 10) in a good body condition and health status. The sampling included ewes in different physiological conditions, i.e., cyclic (*n* = 10), pregnant (*n* = 10), and during their early lactation period (*n* = 10). At the Agris experimental farm, lambs, rams, and ewes in different reproductive status are kept in separated pens according to their productive status. Before sampling, the reproductive status of cyclic and pregnant ewes was confirmed by ultrasound scanning of the ovaries, which was performed with a real-time B-mode scanner (Aloka SSD 500; Aloka Co., Tokyo, Japan), as previously described (38). Ewes in their early lactation period (15–20 days from parturition) were with their lambs in the group of milked ewes. Before starting to collect feces, animals from each experimental groups were moved in deep cleaned pens. Each animal was crayon marked in rump and head using different colors to allow individual identification and avoid double sampling from the same animal. Fecal samples were recovered after posting and observation of the animal's defecation, without causing animal stress.

Sampling was performed on the same day (December 20th, 2021) in all the animals to limit the variations in FTMs due to the external environmental conditions.

The fecal samples were collected with latex gloves and were put into sterile centrifuge tubes, sealed, marked, and transported on ice to laboratory within 2 h from collection.

### FTMs extraction and ELISA assay

0.2 g of fresh feces were individually homogenized and freeze-dried in 15 mL tubes. Then, FTMs were extracted following a protocol previously described by Pasciu et al. (35).

Briefly, the freeze-dried samples were extracted with ethanol 70%, dried under a stream of compressed air and the residues were reconstituted with 1 ml of phosphate buffered saline.

FTMs were analyzed with T3 ELISA kit using a microplate reader (POLARstar Omega; BMG Labtech), with BMG Labtech software for data analysis. The assay is a competitive method designed for human T3 in blood serum and recently validated for mouflon FTMs (35). This kit is based on group-specific polyclonal antibodies able to identify the parent compound and its metabolites. The lower detection limit of the kit (0.05 ng/mL) was suitable for our analytical purpose. The specific cross-reactivity supplied by the manufacturer for the T3 antibody to other compounds was: l-triiodothyronine 1.00%, d-triiodothyronine 0.015%, l-thyroxine 0.01%, d-thyroxine 0.0025%, monoido-tyrosine n.d., diiodo-tyrosine n.d., triiodothyroacetic acid n.d., and tetraiodothyroacetic acid n.d. Quality controls provided by the manufacturer were used to verify the performance of the assay. T3 concentration is calculated through a calibration curve (0–7.5 ng/mL).

This assay kit has been validated analytically and biologically for sheep fecal matrix.

### Analytical validation

For analytical validation, precision, recovery and parallelism were measured. 0.2 g of unfortified feces, obtained from 3 cyclic ewes and 3 rams, were analyzed to determined basal FTMs concentration. To assess recovery and precision parameters, spiked samples were prepared fortifying 0.2 g of fresh feces at two level concentrations (1 and 5 ng/mL of standard T3). Then, all samples were freeze-dried and extracted with ethanol 70% using the method of Pasciu et al. (35). Dry residue was reconstituted with 1 ml of phosphate buffered saline (PBS).

The precision of the method, expressed as percent relative standard deviation (RSD %), was calculated for three replicates in the same day (intra-day repeatability) and over three consecutive days (inter-day repeatability).

The accuracy, which represents the closeness of the test results to the true values, was determined as recovery % for five replicates using the following formula:


$$\begin{array}{l} \frac{FF}{STD}\;\times \;100 \end{array}$$

Where FF were feces fortified, and STD were corresponding T3 standard solutions (1 or 5 ng/mL in PBS).

Parallelism, which allow to verify whether the assay maintains linearity under dilution of unfortified samples (39), was analyzed using serially diluted fecal extracts from 5 real samples (no spiked) at high concentration. In the Elisa test, the purpose of the parallelism study is to verify that the binding of the endogenous analyte to antibodies is the same as the calibrator. At least three serial dilutions were made for samples, and the samples were analyzed in duplicates in the same run, by compensating with the dilution factor. Parallelism was expressed as coefficient of variation (% CV). The % CV <20% indicates the presence of parallelism (39).

### Biological validation

To perform the biological validation of the method, we compared differences in FTM levels between cyclic (*n* = 10), pregnant (*n* = 10) and early lactating ewes (*n* = 10), rams (*n* = 10), and lambs (*n* = 10) collected in the same day (December 20th, 2021): (Average Ambient Temperature = 14.0°C T min = 7.0°C T max = 11.0°C). FTMs values were expressed as ng/g fresh feces. The concentrations obtained ng/mL from the ELISA assay were converted in ng/g wet feces, considering that 0.2 g wet feces (freeze-dried, extracted, and dried) were re-suspended with 1 mL of PBS, obtaining 0.2 g of wet feces in 1 ml of solvent (ng FTMs/g wet feces = FTMs ng/mL/0.2 g/ml).

### Statistical analysis

Results are expressed as mean values (mean ± SE). Variable normality of the studied groups was assessed by the Kolmogorov–Smirnov test. Differences were considered to be statistically significant at *p* < 0.05.

Differences between lambs, rams, and ewes were analyzed by Kruskal–Wallis, using groups as factor, in order to point out statistical differences. Differences between cycling, pregnant and early lactation ewes were analyzed by a One-way ANOVA, using groups as factor, in order to point out statistical differences. As *post-hoc* test, Fisher LSD test was used to highlight possible differences within and between groups.

Analyses were performed using Minitab 17 Statistical Software (2010, Minitab, Inc., State College, PA, USA).

## Results and discussion

Previous researches demonstrated that T3 metabolites can be measured successfully in fecal matrix of wild ungulates (32, 35, 36). This study aims to validate the use of an human serum T3 ELISA kit in sheep feces, as previously described in mouflon (35). The biological validation was performed evaluating FTMs concentrations in sheep in different physiological stages.

The analytical validation on fecal sheep samples showed a good accuracy with a mean recovery of 80.99 and 70.08% at 1 and 5 ng/mL, respectively (Table 1). Recovery rates were acceptable according to international guidelines (70–120%) (40). The repeatability data were within 15%, as requested by the guidelines for method validation (Table 1) (41).

**Table 1:** Repeatability and recovery in fecal samples of sheep.

**Sample**	**T3 concentration**	**Repeatability (RSD%)**		**Recovery (% ±SD)**
		**Intra-Day**	**Inter-Day**	
Feces	1.0 ng/mL	6.42	10.04	80.99 ± 8.93
	5.0 ng/mL	7.89	14.37	70.08 ± 9.04

RSD%, relative standard deviation.

**Figure 1:**
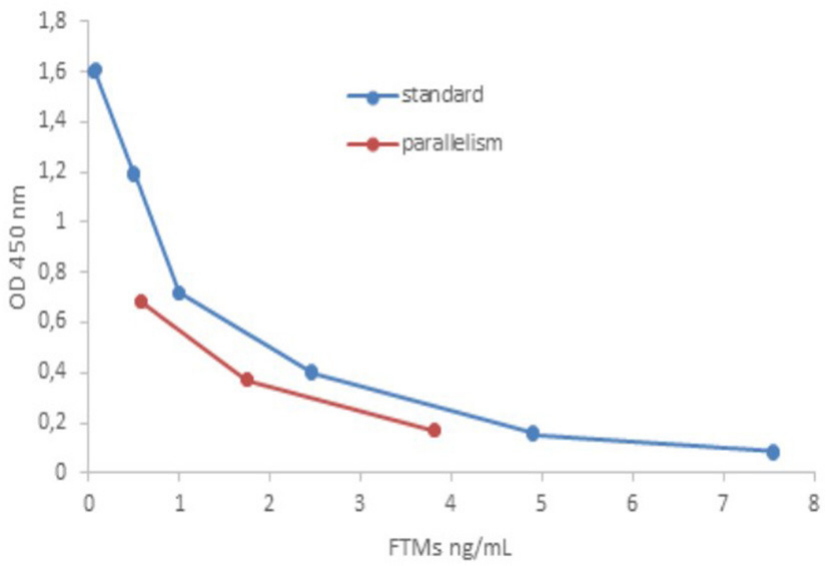
Comparison of standard curve vs. parallelism graphs.

Parallelism, evaluated by dilution of unfortified samples with high endogenous concentrations of the analyte, showed % CV lower than 10% (7.72 ± 2.30), perfectly in accordance with requested values (% CV < 20%) (39). In Figure 1 reported standard curve vs. parallelism graphs was reported (42).

These results confirmed that human serum T3 ELISA kit was suitable for FTMs determination in sheep.

**Figure 2:**
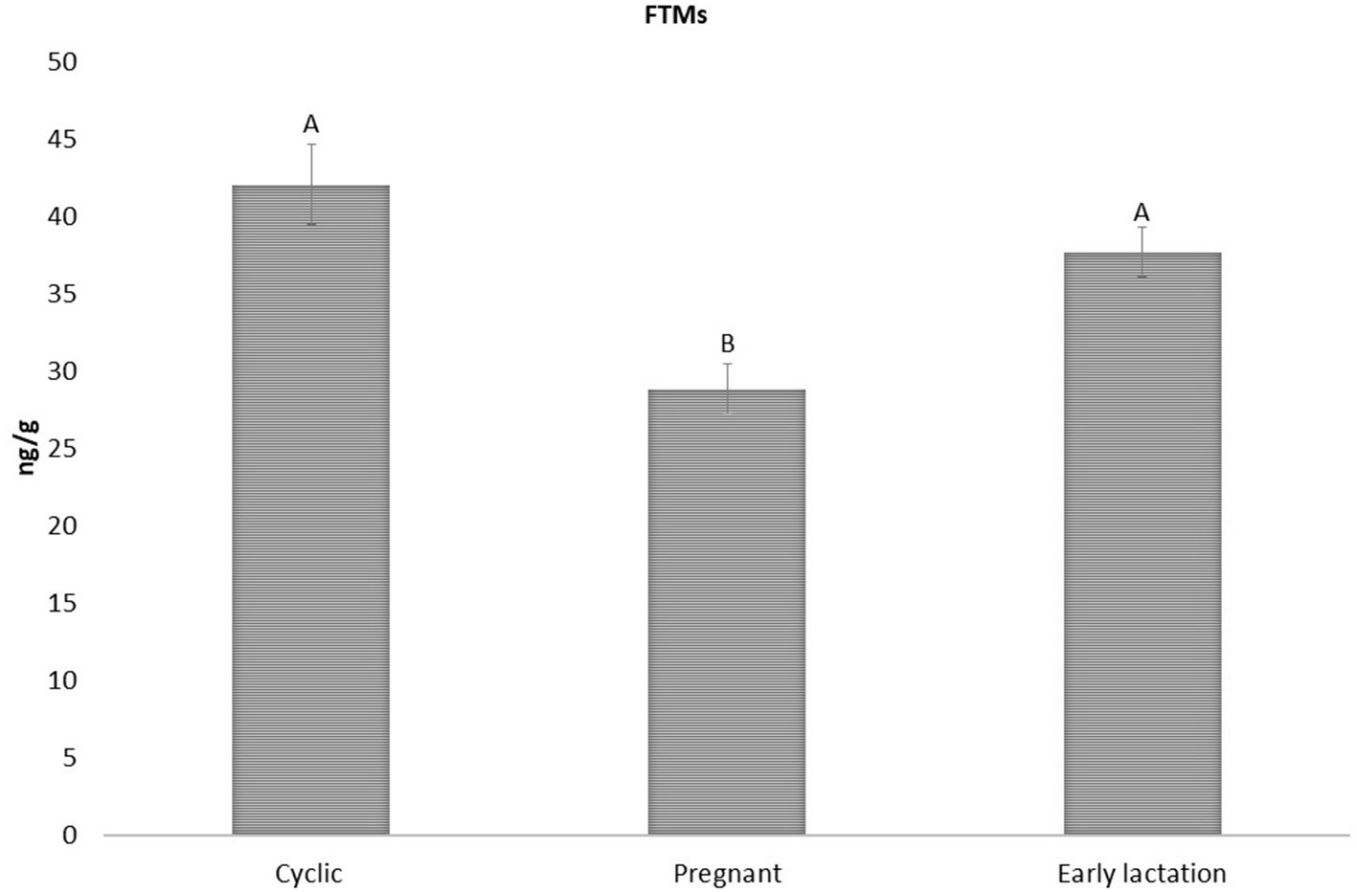
FTMs levels in cyclic early lactation and pregnant sheep (42.07 ± 2.59; 37.70 ± 1.61; and 28.84 ± 1.61 ng/g wet feces, respectively). Upper case letters indicated significant differences between groups (*p* < 0.05).

**Figure 3:**
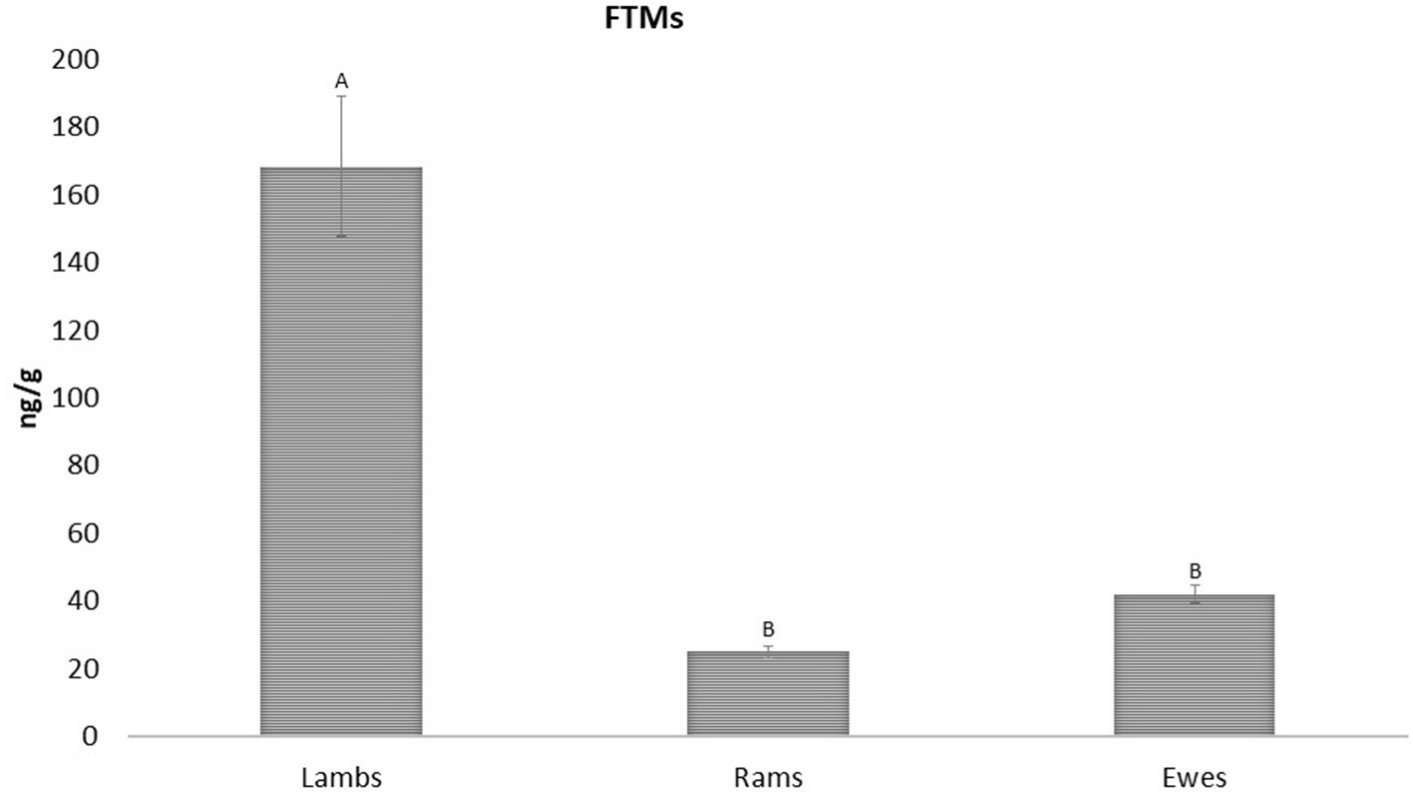
FTMs levels between ewes, rams and lambs (42.07 ± 2.59; 25.03 ± 1.63; and 168.50 ± 20.50 ng/g wet feces, respectively). Upper case letters indicated significant differences between groups (*p* < 0.001).

Considering these results, the method was applied for biological validation in sheep feces, assaying FTM levels at different reproductive status in ewes at first (Figure 2), and then considering variations related to age and sex (Figure 3). As shown in Figure 2, there were no significant differences between FTMs concentration in cyclic (42.07 ± 2.59 ng/g wet feces) and early lactation ewes (37.70 ± 1.61 ng/g wet feces). However, in pregnant ewes, FTMs (28.84 ± 1.61 ng/g wet feces) were significantly lower (*p* > 0.05) than in the other two groups. In mammalian species, THs are essential for the maintenance of the female reproductive behavior (e.g., sustain pregnancy and raise offspring) (32). Late pregnancy and lactation are the two reproductive periods with high energetic demand (43). Shi et al. reported that, in antelope, fecal T3 concentration was significantly higher in the postpartum period compared to late pregnancy (25). In fact, during lactation, extra energy is needed for milk production (31). Furthermore, several studies carried out on Sudanese and Chinese women, showed that the T3 levels were significantly lower in pregnant compared to non-pregnant women and T3 levels decreased during pregnancy (44, 45). These findings are in line with our results on FTMs differences between pregnant, cyclic, and early lactation ewes (Figure 2).

Regarding sex and age, in this study there were no significant differences in FTMs levels between ewes and rams (42.07 ± 2.59 and 25.03 ± 1.63 ng/g wet feces, respectively), but higher values were found in lambs when compared to adults (168.50 ± 20.50 ng/g wet feces) (*p* < 0.001; Figure 3).

The lack of differences found between sexes confirms the data reported in our previous work in mouflons (35), notwithstanding other studies reported in literature have controversial outcome: some authors reported that females ungulate have higher THs values than males (32, 46), while other reports opposite results (47, 48). Finally, other authors described no difference between sexes (33, 34, 49). As well as sex, also the age can affect THs levels, because of their involvement in the developmental processes, and levels are higher after birth and during the growth period, declining thereafter (50). In fact, in literature is described that young animals have higher THs levels than adults (12, 32, 48, 51). For wild ungulates (forest musk deer Moschus berezovskii), Hu et al. (32) reported that fecal T3 concentrations differ among age groups; in particular, younger individuals showed FTMs concentrations higher than adults, and, among young, as they grow up, the FTMs levels decreased. The main advantage of this study is that it provides useful information about FTMs levels in different life history stages (e.g., development or pregnancy), suggesting how different energy requirement can vary according to their respective needs. Furthermore, another advantage is the possibility of using a non-invasive matrix, which is very important for animal welfare. A limitation of this work is the lack of repeated sampling for each experimental group, which would have strengthened the biological validation. This is partly overcome by the number of animals used per experimental group and by the fact that the reproductive status was known and confirmed by ultrasound scanning of the reproductive tract. In addition, having determined thyroid hormone concentrations also in blood would have confirmed the difference found. This will be a possible goal for future studies. Furthermore, future studies could provide more information on energy expenditure during the different production stages of farm animals by considering the details on the fraction of calories introduced and on energy expenditure. Moreover, it could be interesting to carry out studies on fecal T3 variations depending on seasonality, environmental temperature, type of alimentation, as well as parasitic infections and alteration in intestinal microbiota. In the livestock sector, it could allow to improve our knowledge about the response of animals to different physiological and environmental conditions, and thus assess their welfare.

## Conclusion

In conclusion, in this study, we have established that FTMs can be reliably and accurately determined in sheep feces, using an ELISA kit formulated for human serum T3 assay. The application of the validated method on feces of cyclic, pregnant, and early lactating ewes as well as in rams and ewe lambs, provides valuable information on the physiological state of this specie.

The results found show how the use of a non-invasive method for the animal and a readily available matrix such as feces, can represent a useful tool not only for the study of the physiological conditions in sheep, but also for future studies on energy expenditure, seasonality, and disease conditions.

## Data availability statement

The raw data supporting the conclusions of this article will be made available by the authors, without undue reservation.

## Ethics statement

Ethics approval was not required for the study on animals because all procedures carried out were non-invasive. Any invasive procedures were carried out during our previous study (38), which was approved by the Italian Ministry for Health (n. 705/2019-PR).

## Author contributions

VP, MN, and FB designed the study. CP collected fecal samples. VP and FDS performed the experiments. VP prepared the manuscript. VP, MN, CP, and FB analyzed the data. CP, FDS, MN, and FB revised the manuscript. All authors contributed to the article and approved the submitted version.

## Funding

This work was funded by the University of Sassari (Fondo di Ateneo per la ricerca 2020), Attrazione Mobilitàdei dei Ricercatori (PON AIM 1887720-1 CUP J54I18000160001), and Ricerca e innovazione 2014-2020 (PON DM1062MEDVET-IV6).

## Conflict of interest

The authors declare that the research was conducted in the absence of any commercial or financial relationships that could be construed as a potential conflict of interest.

## Publisher's note

All claims expressed in this article are solely those of the authors and do not necessarily represent those of their affiliated organizations, or those of the publisher, the editors and the reviewers. Any product that may be evaluated in this article, or claim that may be made by its manufacturer, is not guaranteed or endorsed by the publisher.
